# *Ehrlichia chaffeensis* TRP120 nucleomodulin binds DNA with disordered tandem repeat domain

**DOI:** 10.1371/journal.pone.0194891

**Published:** 2018-04-11

**Authors:** Valerie J. Klema, Krishna Mohan Sepuru, Nadia Füllbrunn, Tierra R. Farris, Paige S. Dunphy, Jere W. McBride, Krishna Rajarathnam, Kyung H. Choi

**Affiliations:** 1 Department of Biochemistry and Molecular Biology, Sealy Center for Structural Biology and Molecular Biophysics, University of Texas Medical Branch, Galveston, Texas, United States of America; 2 Department of Pathology, Center for Biodefense and Emerging Infectious Diseases, Sealy Center for Vaccine Development, University of Texas Medical Branch, Galveston, Texas, United States of America; Russian Academy of Medical Sciences, RUSSIAN FEDERATION

## Abstract

*Ehrlichia chaffeensis*, the causative agent of human monocytotropic ehrlichiosis, secretes several effector proteins that bind host DNA to modulate host gene expression. The tandem repeat protein 120 (TRP120), one of the largest effector proteins, has four nearly identical tandem repeat (TR) regions that each consists of 80 amino acids. In addition to playing a role in ehrlichial binding and internalization, TRP120 translocates to the host nucleus where it is thought to function as a transcription factor that modulates gene expression. However, sequence analysis of TRP120 does not identify the presence of DNA-binding or trans-activation domains typical of classical eukaryotic transcription factors. Thus, the mechanism by which TRP120 binds DNA and modulates gene expression remains elusive. Herein, we expressed the TR regions of the TRP120 protein, and characterized its solution structure and ability to bind DNA. TRP120, expressed as either a one or two TR repeat, is a monomer in solution, and is mostly disordered as determined by circular dichroism (CD) and nuclear magnetic resonance (NMR) spectroscopy. Using NMR spectroscopy, we further show that the 1 TR construct selectively binds GC-rich DNA. Although low pH was required for TRP120 TR-DNA interaction, acidic pH alone does not induce any significant structural changes in the TR region. This suggests that TRP120 folds into an ordered structure upon forming a protein-DNA complex, and thus folding of TRP120 TR is coupled with DNA binding.

## Introduction

*Ehrlichia chaffeensis*, the etiologic agent of human monocytotropic ehrlichiosis (HME), is an obligate intracellular bacterium and a member of the *Anaplasmataceae* family [1, 2]. Transmitted by the lone star tick (*Amblyomma americanum)*, *E*. *chaffeensis* infects human mononuclear phagocytes where it replicates forming microcolonies in cytoplasmic membrane-bound vacuoles called morulae [3]. HME presents symptoms ranging from mild febrile illness, malaise, and headache, to life-threatening septic shock, respiratory distress, and renal or hepatic failure in more serious cases, with over half requiring hospitalization and 3% proving fatal [4, 5]. HME patients exhibit severe manifestations despite a low bacterial burden, suggesting a role for immunopathologic mechanism in disease pathogenesis [6, 7]. However, because the symptoms of *E*. *chaffeensis* infection are similar to other undifferentiated febrile illnesses and tick-borne infections endemic to the same geographic areas, HME is often misdiagnosed and underreported [8].

In order to survive intracellularly, *E*. *chaffeensis* reprograms the host cell by activating host cell pathways such as Wnt and Notch, and by secreting several tandem repeat protein (TRP) effectors into the host cell via a type I secretion system. Ehrlichial TRP effectors interact with an array of host cell proteins in order to exploit and modulate host cell processes. *E*. *chaffeensis* TRP120 and TRP32 are known to translocate to the nucleus where they bind genes associated with immune regulation, post-translational modifications, transcriptional and translational regulation, signal transduction, apoptosis, and metabolism [9]. In particular, TRP32 has been shown to bind promoter regions in the host DNA to repress or activate host gene transcription [9, 10]. Although ehrlichial TRP120 and TRP32 lack a classical nuclear localization signal sequence, nuclear translocation appears to be mediated in part by post-translation dependent modifications. Nuclear translocation of TRP32 has been shown to proceed via a tyrosine phosphorylation-dependent mechanism [10]; however, the mechanism of TRP120 nuclear translocation is unknown [9, 10]. Interestingly, *Ehrlichia* TRPs exhibit similarity to the *Xanthomonas* and *Ralstonia* species transcription activator-like (TAL) effectors, which utilize similar tandem repeat DNA binding domains to activate plant cell gene transcription [11, 12].

**Fig 1:**
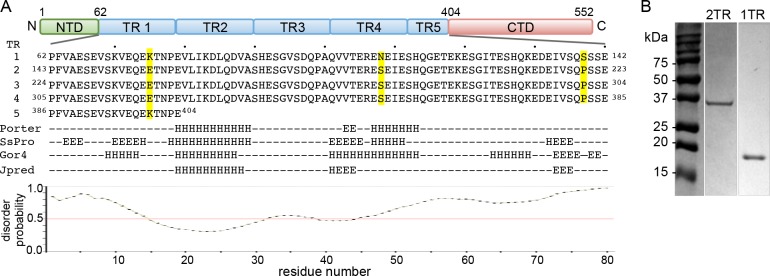
TR units of TRP120. (A) Domain organization of TRP120 and sequences of each TR unit. The primary sequences of TR units are shown with residues that differ between TR units highlighted in yellow (top). The sequence was analyzed by secondary structure (middle) and disorder (bottom) predictions. Predicted α-helical and β-strand regions are indicated by H and E, respectively. (B) SDS-PAGE of purified TRP120-1TR and TRP120-2TR proteins. Molecular weights for TRP120-1TR and -2TR proteins are 11.5 and 20.3 kDa, respectively, but the proteins migrate at twice their expected size.

*E*. *chaffeensis* TRP120 consists of a small N-terminal domain of unknown function, a central TR repeat region, and a C-terminal domain (Fig 1A). The C-terminal domain is sumoylated at a canonical motif and also has HECT E3 ligase activity [13]. The central region consists of four 80 amino acid TR units and a fifth shortened repeat containing 19 residues. The TR units within TRP120 are 96% identical at the amino acid level, with only three variable amino acids at positions 15, 48, and 77 (Fig 1A). *E*. *chaffeensis* TRP120 binds a GC-rich DNA motif that is similar to GC-rich motifs bound by eukaryotic transcription factors, suggesting that TRP120 functions similarly [9]. Two full repeats of the TRP120 TR unit are sufficient to bind a double-stranded DNA (dsDNA) and oligonucleotide probes containing GC-rich motifs [9]. However, sequence analysis of TRP120 does not identify structural motifs characteristic of typical DNA-binding proteins. Thus, the mechanism by which TRP120 recognizes DNA and functions as a transcription factor has remained elusive. Neither the minimum TRP120 sequence nor the conditions required for DNA binding are well understood. Furthermore, TRP120 contains four copies of nearly identical repeats, but their influence on the structure and function of TRP120 has not been determined. In this study, we demonstrate that TRP120 containing, either a single or two TR units, exist mostly as intrinsically disordered monomers, and that a single TR unit is sufficient to bind DNA. The interaction between the TRP120 TR unit and GC-rich DNA is pH-dependent, but pH alone does not induce structural changes in the TR unit. Folding of TRP120 TR requires both low pH and interaction with DNA, and thus TRP120 undergoes a coupled folding and binding process.

## Materials and methods

### Generation of TRP120-1TR construct

Construction of the glutathione *S*-transferase (GST)-fused TRP120 containing two TR units (TRP120-2TR) is previously described [9]. TRP120 protein containing a single TR unit (TRP120-1TR) was generated as a GST-fusion protein. To produce TRP120-1TR, the TRP120-2TR construct within the pGEX expression vector (TRP120-2TR) used in previous studies served as a template [9]. The oligonucleotide primers 5’-TTAGAAACTTCACTTTCTGCAACAAATTACTCTGATGAAGATTGAGATACTATTTC-3’ (forward) and 5’-GAAATAGTATCTCAATCTTCATCAGAGTAATTTGTTGCAGAAAGTGAAGTTTCTAA-3’ (reverse) were used with the QuikChange II site-directed mutagenesis kit (Agilent Technologies) to isolate the second of the two TR units in the template. The sequence of the final TRP120-1TR construct was confirmed by DNA sequencing at the Molecular Genomics Core at the University of Texas Medical Branch. The final TRP120-1TR contains a single TR region and 22 residues from the thrombin cleavage site and N-terminal domain (Gln47-Glu61).

### Expression and purification of TRP120 constructs

The TRP120-1TR and TRP120-2TR constructs were purified as previously described with minor changes [14, 15]. Briefly, 5 mL of overnight culture grown in Luria Broth were used to inoculate 4 L of Terrific Broth supplemented with 34 μg/ml chloramphenicol and 100 μg/ml ampicillin. The cells were grown at 37°C to an OD_600_ of ~0.8, and protein expression was induced with 0.5 mM isopropyl-β-D-thiogalactopyranoside (IPTG) at 18°C overnight. The harvested cell pellet was sonicated in 60 mL lysis buffer (10 mM phosphate, pH 7.3, 138 mM NaCl, and 2.7 mM KCl) supplemented with 100 μg/ml DNase A and 2 tablets of protease inhibitor cocktail (Roche Applied Science). The GST-fused TRP120 protein was bound to pre-equilibrated Glutathione Sepharose 4B resin (GE Healthcare) at 4°C overnight. The resin was washed three times in 60 mL wash butter (20 mM Tris pH 7.0, 300 mM NaCl, and 1 mM DTT). The TRP120 protein was cleaved from the resin-bound GST tag by incubation with thrombin (1U/mg protein) from bovine pancreas (Sigma) at room temperature for 2 h. The eluted protein was buffer exchanged depending on the relevant experiment. Because the TRP120-1TR and -2TR constructs do not contain any Trp residues, the protein concentration was measured using the Bradford assay with lysozyme (MP Biomedicals) as the protein standard.

To express ^15^N-labeled TRP120-1TR, cells from 1 mL of overnight culture in Luria broth were harvested and resuspended in 50 mL M9 medium supplemented with 1 mg/L ^15^NH_4_Cl (Cambridge Isotope Laboratories), 34 μg/mL chloramphenicol and 100 μg/mL ampicillin. This intermediate culture was grown at 37°C for ~6 h before inoculation of 1 L M9 medium supplemented with 1 mg/L ^15^NH_4_Cl, 34 μg/mL chloramphenicol and 100 μg/mL ampicillin. The expression and purification of ^15^N-labeled TRP120-1TR using affinity chromatography proceeded as described above.

### Sedimentation velocity analytical ultracentrifugation

TRP120-1TR and TRP120-2TR constructs were subjected to sedimentation velocity analytical centrifugation using a Beckman Coulter XL-A analytical ultracentrifuge. To obtain an absorbance reading ~0.7 at a wavelength of 224 nm, proteins were diluted in 20 mM Tris (pH 7.0), 300 mM NaCl, and 1 mM DTT to a concentration of 27 μM. Protein sedimentation was carried out at 50,000 rpm at a temperature of 20°C and 350 absorbance scans were collected. Data were analyzed using the program SEDFIT [16]. During data processing, the size distribution model “*c(s)* continuous” was selected and the frictional ratio was allowed to float.

#### Sequence analysis

The sequence of TRP120 was analyzed by the ExPASy bioinformatics resource portal [17]. The program ProtParam [18] was used to determine the molecular weights and isoelectric points. The secondary structure prediction programs Jpred [19], SsPro [20], Porter [21], and Gor4 [22] were used to predict the secondary structure content of a single 80 amino acid TR unit. Disordered region prediction programs PrDOS [23], IUPred [24], DisEMBL [25], PredictProtein [26] were used to predict the disordered regions in TRP120.

### Circular dichroism spectroscopy of TRP120 constructs

Far-UV circular dichroism (CD) spectroscopy was used to determine the secondary structure content of the TRP120-1TR and TRP120-2TR constructs. CD data were collected using 9 μM TRP120-1TR in 20 mM Tris buffer (pH 7.0) containing 100 mM NaCl. To monitor changes in TRP120-2TR secondary structure in different pH buffers, TRP120-2TR in 10 mM phosphate (pH 7.0) and 100 mM NaCl was buffer exchanged into 10 mM phosphate (pH 6.0) and 100 mM NaCl, or 10 mM citrate (pH 4.5) and 100 mM NaCl. CD spectra were measured at 20°C from 195 nm to 250 nm by a Jasco J-815 spectrometer using a data integration time of 8 sec and were averaged over 3 replicates. Data were converted from millidegrees to molar residue ellipticity (MRE) using the formula:

$$\left[\theta \right]=\left(\theta \;\times 10^{6}\right)/\left(c\times \ell \times n\right)$$

where [*θ*] is the molar residue ellipticity, *θ* is the CD signal in millidegrees, *c* is the protein concentration in μM, *l* is the pathlength in mm, and *n* is the number of residues. The CD data analysis program BestSel was used to analyze the data and estimate the secondary structure [27].

### 2D NMR spectroscopy of TRP120-1TR

Complementary DNA oligonucleotides containing the TRP120 GC-rich DNA binding motif 5’-GCGATTCTCCTGCCTCAGCCTCCCTAG-3’ and 5’-CTAGGGAGGCTGAGGCAGGAGAATCGC-3’ were synthesized by Integrated DNA Technologies (Coralville, IA). As a negative control, complementary AT-rich oligonucleotides, 5’-GTCACAAATTCTCATCTTTATATAAAGAT-3’ and 5’-ATCTTTATATAAAGATGAGAATTTGTGAC-3’ were also synthesized. The DNA probes were mixed in a 1:1 ratio before being denatured at 95°C for 10 minutes and annealed by slow cooling to room temperature. HSQC spectra of ^15^N-labeled TRP120-1TR were collected using the protein prepared at pH 7.0 (20 mM Tris), pH 6.5 (10 mM phosphate), pH 6.0 (10 mM phosphate), and pH 5.5 (10 mM phosphate) containing 100 mM NaCl. NMR titrations of DNA to ^15^N-TRP120-1TR were carried out in 20 mM Tris (pH 7.0) or 10 mM sodium phosphate (pH 5.5) containing 100 mM NaCl, 1 mM DSS (2,2-dimethyl-2 silapentanesulfonic acid), 1 mM sodium azide, and 10% D_2_O (v/v). NMR spectra were acquired at 25°C on a Bruker Avance III 800 MHz (equipped with a TXI cryoprobe) or 600 MHz (with QCI probe) spectrometers. Aliquots of DNA (~ 10 mM) prepared in the same buffer were added to ~60 μM TRP120-1TR at a 1, 5 or 10-fold molar excess, and a series of ^1^H-^15^N HSQC spectra was collected.

## Results

### TRP120-1TR and TRP120-2TR exist as monomers in solution

**Fig 2:**
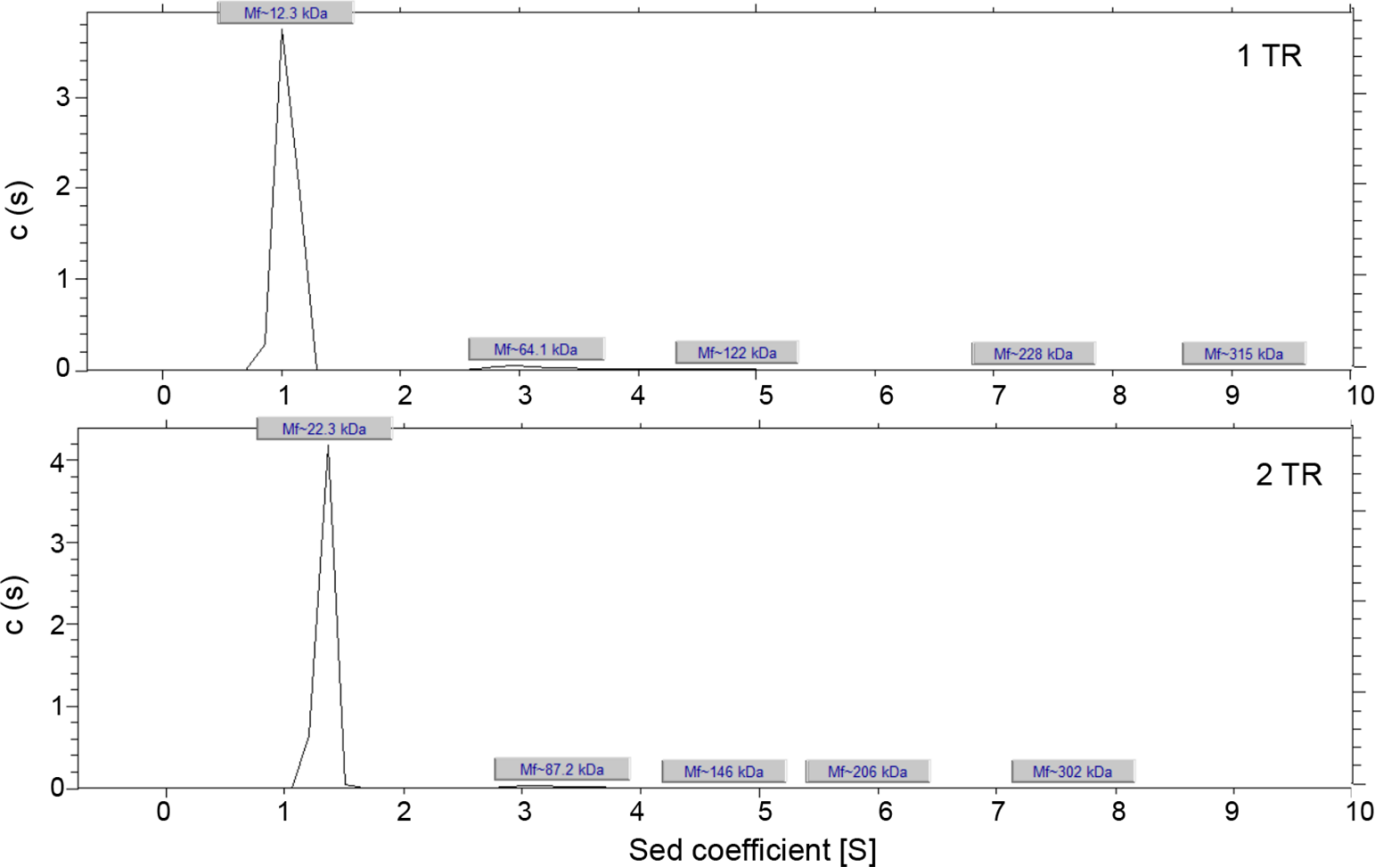
Sedimentation velocity profiles of TRP120-1TR and TRP120-2TR proteins. The molecular weights of TRP120-1TR and -2TR proteins were determined by sedimentation velocity experiments. Sedimentation coefficient distribution *c(s)* profile for TRP120-1TR and TRP120-2TR show one major species, which corresponds to the molecular weight of 12.3 or 22.2 kDa, respectively. The observed molecular weights indicate that both TRP120 constructs are monomers in solution.

TRP120 protein consists of an N-terminal domain, 4.3 TR units, and a C-terminal domain (Fig 1A). The 4.3 repeats of the TR unit (339 amino acids) form the majority of the protein sequence. At this time, little is known regarding the structural properties of the TR units, whether the TR units bind individually or if crosstalk between the domains is necessary for folding and DNA binding. To address these questions, we characterized two TRP120 TR constructs–one containing a single TR unit (80 residues, TRP120-1TR) and the second containing two TR units (TRP120-2TR). TRP120-1TR and TRP120-2TR were expressed in *E*. *coli* as GST-tagged fusion proteins and purified via affinity chromatography to a purity >95% as judged by SDS-PAGE (Fig 1B). Full-length TRP120 is a 60 kDa protein, but migrates as a ~120 kDa protein on SDS-PAGE, twice its predicted molecular weight. Similarly, although the predicted molecular weights of TRP120-1TR and -2TR based on their primary sequences are 11.5 kDa and 20.3 kDa, respectively, the constructs migrate with apparent molecular weights of ~19 kDa and ~36 kDa, respectively (Fig 1B). To determine if TR regions form a dimer that is stable during SDS-PAGE, the oligomeric states of the TRP120 constructs were examined by sedimentation velocity analytical ultracentrifugation. TRP120-1TR and -2TR constructs exhibited sedimentation coefficients of 1.1S and 1.4S, corresponding to molecular weights of 12.3 kDa and 22.3 kDa, respectively (Fig 2). Thus, both proteins are monomeric in solution. The frictional ratios determined during data processing were 2.0 and 2.2 for TRP120-1TR and TRP120-2TR, respectively, indicating a significantly elongated or asymmetric shape.

The anomalous migration of TRP120 in SDS-PAGE was determined to be related to the highly acidic pI of the protein [28]. TRP120 has an isoelectric point (pI) of 4.1 for the full-length protein, and 3.7, 4.3, and 4.1 for the individual N-terminal, central TR and C-terminal domains, respectively. We found that the TRP120-1TR and -2TR proteins expressed in *E*. *coli* also migrate to higher molecular weights than expected. The analytical ultracentrifugation clearly shows that both TRP120-1TR and -2TR proteins are monomers with expected molecular weights. Thus, aberrant migration of TRP120 TR units on SDS-PAGE is intrinsic to the nature of the domain. The single TRP120 TR unit contains 16–17 Glu and 4 Asp residues out of 80 amino acids (Fig 1A), and thus the highly acidic TRP120 TR proteins resist SDS binding and migrate as a higher molecular weight than expected [29] [30].

### TRP120-1TR and TRP120-2TR proteins are mostly disordered in solution

**Fig 3:**
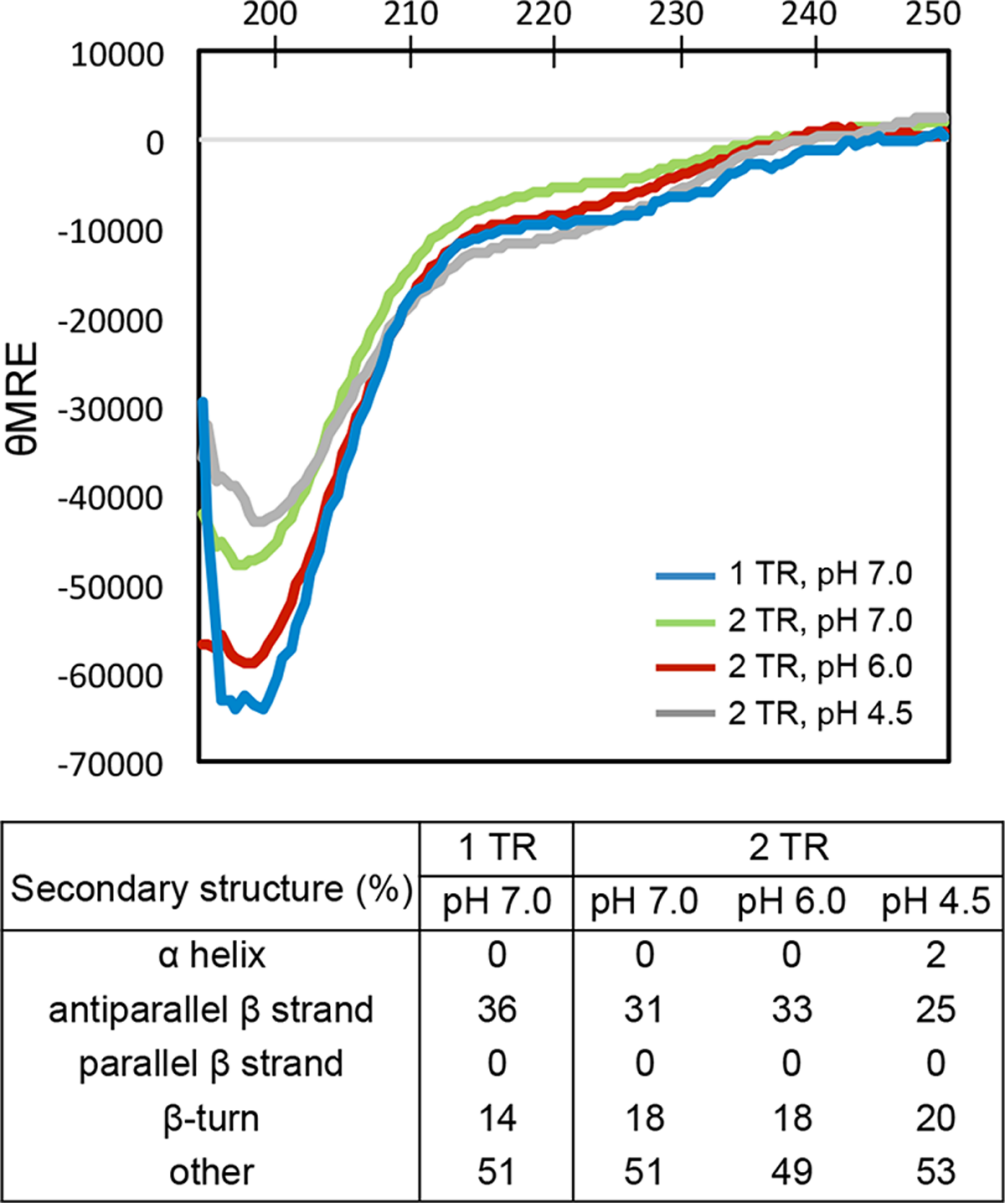
Far-UV CD spectra of TRP120-1TR and TRP120-2TR. The CD spectrum of TRP120-1TR was measured in Tris pH 7.0 containing 300 mM NaCl (blue). The CD spectra of TRP120-2TR were measured at three different pHs—phosphate pH 7.0 (green), phosphate pH 6.0 (red), and citrate pH 4.5 (gray) containing 100 mM NaCl. Secondary structure content of TRP120 constructs was estimated by BestSel analysis (bottom).

Secondary structure prediction programs [19, 20, 22, 31] predict that the TRP120 TR region of 80 amino acids consists of mostly α-helical structure (Fig 1A). However, disorder prediction programs suggest that TR region may be disordered, consistent with the elongated shapes of the TRP120 TR constructs indicated by analytical ultracentrifugation (Fig 1A). We thus determined the secondary structure content of TRP120-1TR and -2TR by CD. The far-UV CD spectra of TRP120-1TR and TRP120-2TR at pH 7.0 showed similar profiles, with a minimum centered at 200 nm, indicating that TRP120 TR units are mostly intrinsically disordered (Fig 3). A shallow negative feature centered at 220 nm suggests a small percentage of β-turn/β-sheet secondary structure. The program BestSel was used to analyze the CD data, and the program estimated ~50% random coil and 31~36% antiparallel β-strand content for both proteins (Fig 3). In contrast to the secondary structure prediction, no α-helices were observed. Additionally, we measured the CD spectra of TRP120-2TR at lower pH ranges, because the pH of ehrlichial morulae where TRP120 is expressed is slightly acidic due to functional vacuolar H^+^ ATPase that acidifies the compartments [32]. CD spectra of TRP120-2TR measured as a function of pH (7.0, 6.0 and 4.5) showed similar profiles except for small changes in the β-strand content (Fig 3). However, these changes are modest and the TRP120-1TR and -2TR proteins are mostly intrinsically disordered.

### TRP120 tandem repeat binds GC-rich DNA

**Fig 4:**
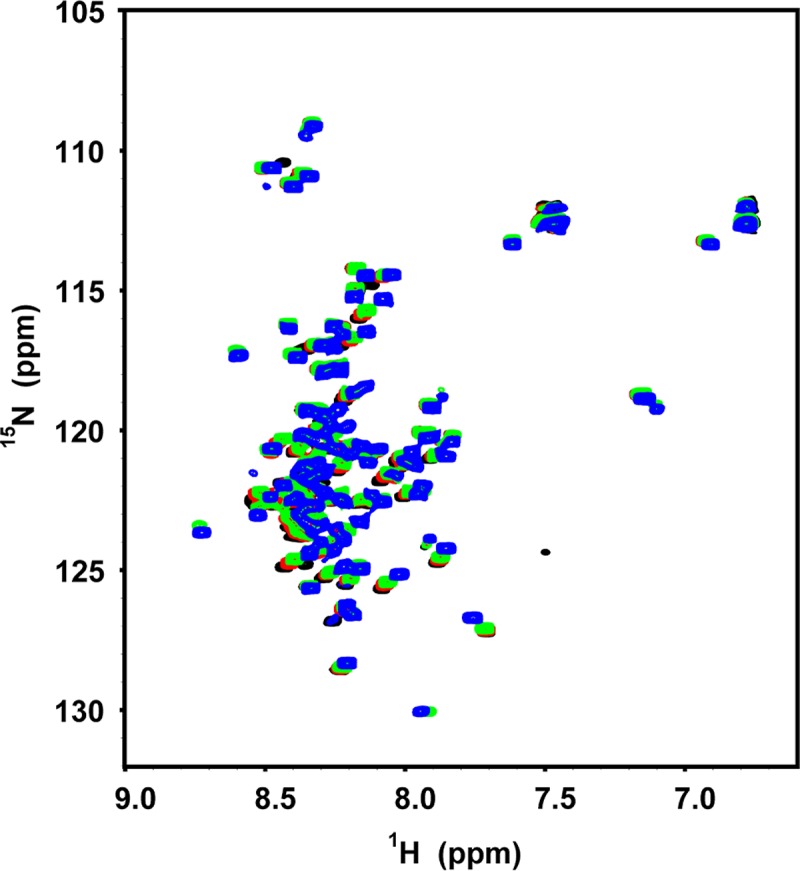
NMR spectra of TRP120-1TR at different pHs. HSQC spectra of ^15^N-labeled TRP120-1TR were collected at four different pHs—Tris pH 7.0 (black), phosphate pH 6.5 (red), phosphate pH 6.0 (green), and phosphate pH 5.5 (blue) containing 100 mM NaCl.

TRP120 is known to modulate gene expression in the host nucleus, and to bind 20–30 nt dsDNA containing a GC-rich region (Table 1) [9]. We characterized the binding of ^15^N-labeled TRP120-1TR to dsDNA to determine if the TRP120 TR region can bind DNA and whether there is a structural change in the TR region upon DNA binding. We first collected ^1^H-^15^N NMR HSQC spectra of TRP120-1TR as a function of pH (5.5, 6.0, 6.5 and 7.0), and observed that the protein is essentially unstructured in solution at all pHs tested (Fig 4), consistent with the CD data. The NMR spectrum shows around 80 resonances between 6.7 and 8.6 ppm (Fig 4). Since the protein consists of 102 residues, most of the residues are accounted for. However, most of the chemical shifts correspond to that of a random coil and are not significantly downfield shifted (characteristic of β-sheet) or upfield shifted (characteristic of α-helix), and thus NMR data are consistent with a natively unfolded protein.

**Table 1:** DNA probes used in NMR titrations.

DNA probe	Sequence
GC-rich*	5’-GCGATTCTCCTGCCTCAGCCTCCCTAG-3’
	3’-CGCTAAGAGGACGGAGTCGGAGGGATC-5’
AT-rich	5’-GTCACAAATTCTCATCTTTATATAAAGAT-3’
	3’-CAGTGGGGAAGAGTAGAAATATATTTCTA-5’

* The GC-rich TRP120-binding motifs are underlined.

**Fig 5:**
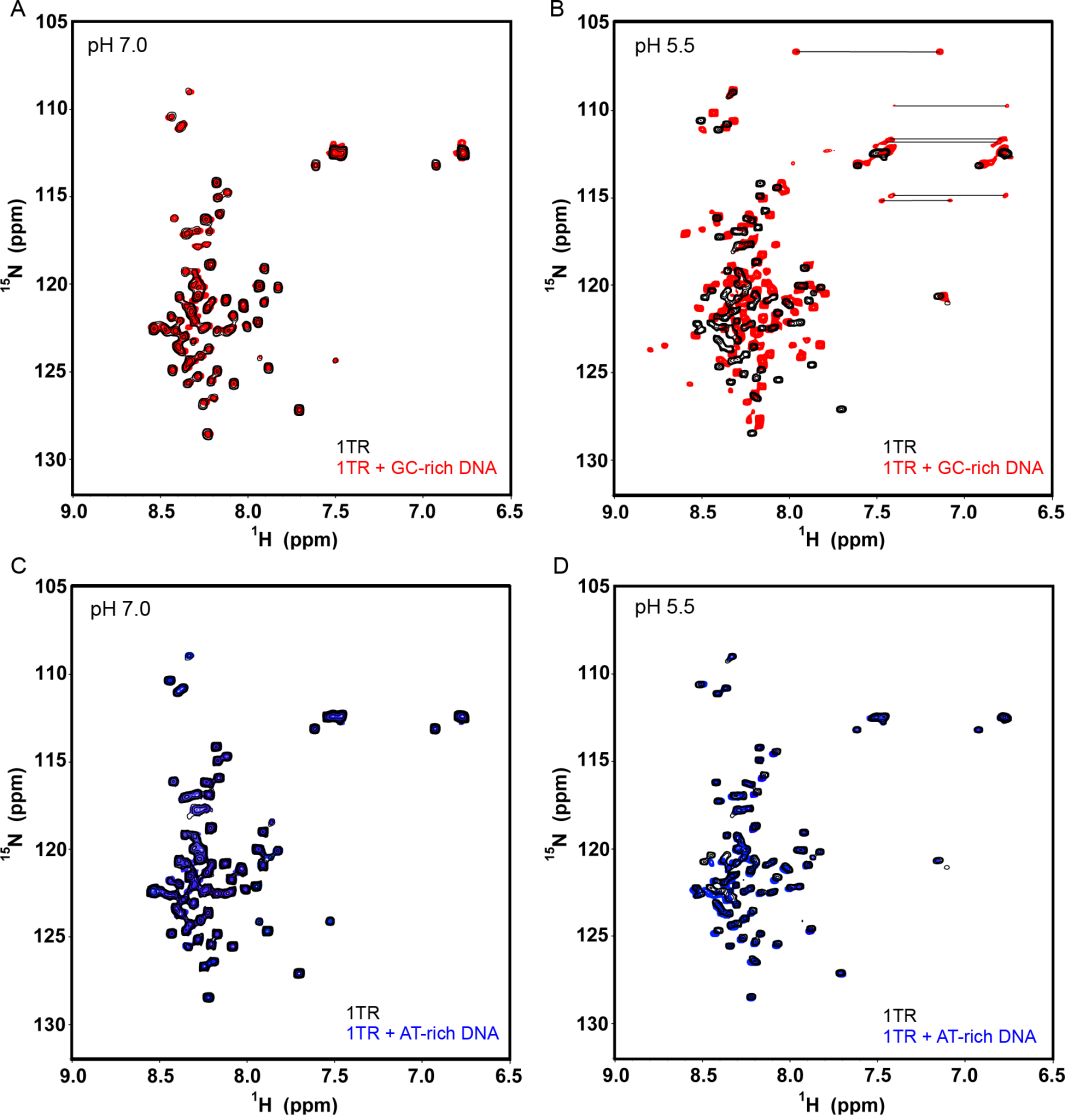
TRP120-1TR selectively binds to GC-rich DNA. (A-B) HSQC spectra of ^15^N-labeled TRP120-1TR with GC-rich DNA at pH 7.0 or pH 5.5. TRP120-1TR (68 μM) alone (black) and with a 10-fold molar equivalent of unlabeled DNA (red, Table 1) in 20 mM Tris pH 7.0 containing 100 mM NaCl or in 10 mM phosphate pH 5.5 containing 100 mM NaCl are shown. The lines highlight Asn or Gln side chain NH_2_ peaks that show distinctly different chemical shifts in the bound form. (C-D) HSQC spectra of ^15^N-labeled TRP120-1TR with AT-rich DNA probe at pH 7.0 or pH 5.5. TRP120-1TR (30 μM) alone (black) and with a 10-fold molar equivalent of unlabeled DNA (blue, Table 1) in 20 mM Tris pH 7.0 containing 100 mM NaCl or in 10 mM phosphate pH 5.5 containing 100 mM NaCl are shown.

Interestingly, when we carried out binding measurements with 27 nt GC-rich DNA at pH 5.5 and 7.0, TRP120-1TR binds DNA at pH 5.5 but not at 7.0. At pH 7.0, the addition of DNA to TRP120-1TR up to 10-fold molar equivalent did not show significant chemical shifts, indicating minimal binding and no induction of secondary structure formation (Fig 5A). However, addition of DNA to TRP120-1TR at pH 5.5 resulted in several new peaks with significant differences in chemical shifts compared to the parent peaks (Fig 5B). The presence of new downfield shifted peaks (>8.5 ppm) suggests an increase in β-strand content. Furthermore, the peaks corresponding to several side chain NH_2_ of Asn/Gln residues were resolved in the DNA-bound spectrum, indicating that these residues become structured upon binding to DNA. Overall, the significant chemical shift perturbation (CSP) and large dispersion in chemical shifts in the DNA-bound spectrum compared to the free TRP120-1TR indicate that the protein adopts a specific structure and conformation upon DNA interaction. In contrast, when the AT-rich oligonucleotide was used in TRP120-1TR binding, there were no significant chemical shifts at pH 7.0, and only a few minor chemical shifts at pH 5.5 (Fig 5C and 5D). Thus, the interaction between TRP120-1TR and the GC-rich oligonucleotide is specific, which would be necessary for observed transcription activities [9].

## Discussion

*E*. *chaffeensis* TRP effectors are involved in the activation of host cell pathways, manipulation of gene transcription, and exploitation of post-translational modifications to reprogram the host cell. TRP120 and TRP32 translocate through the host nuclear membrane via mechanisms that do not involve a classic nuclear localization sequence. A previous study reported that TRP120 binds a GC-rich motif within the host DNA and alters gene expression [9]. However, many of the molecular and structural details of TRP120 DNA binding are unclear, including characterization of structural features, the minimum TRP120 sequence necessary for structural integrity, and conditions necessary for DNA binding. In this study, we demonstrate by CD and NMR spectroscopy that TRP120 TR regions are intrinsically disordered in the absence of DNA. However, in the presence of DNA, a single TR unit of TRP120 binds selectively to GC-rich DNA over AT-rich DNA (Fig 5). Additionally, we show that DNA binding by TRP120 TR is pH-dependent, with virtually no binding at pH 7.0 but binding at pH 5.5. Notably, pH change alone does not trigger any structural changes in TRP120, since the secondary structure content of the protein does not change as a function of pH (Figs 3 and 4).

Intrinsically disordered proteins have been shown to be involved in regulation of transcription and translation, cellular signaling, protein phosphorylation, and self-assembly of large protein complexes [33]. TRP120 shares several features with intrinsically disordered proteins. First, intrinsically disordered proteins have characteristic sequences, typically containing a low proportion of order promoting amino acids (Cys and bulky hydrophobic amino acids) and a high proportion of particular polar and charged amino acids (Gln, Ser, Pro, and Glu) [33]. The TR region of TRP120 does not contain Cys, Met, Tyr, or Trp residues, and contains 17 Glu, 12 Ser, and 7 Gln residues, consistent with its disordered nature (Fig 1). The intrinsic plasticity typical of disordered proteins may enable TRP120 to interact with diverse host targets [34]. TRP120 interacts with an array of host proteins such as immunoglobulin light-chain lambda and polycomb group ring finger 5, involved in immune and cellular signaling, metabolism, and protein synthesis/degradation to control over the immune response and facilitate infection [34]. Second, intrinsically disordered proteins often undergo a coupled folding and binding process, i.e., folding into an ordered structure upon complex formation with their targets [35]. Our data suggest that the conformational change in TRP120-1TR is coupled with DNA-binding, since low pH alone does not induce any structural changes in the TR regions of TRP120 (Figs 4 and 5). Third, intrinsically disordered protein regions generally participate in highly specific but low affinity interactions, which are required due to the transient nature of interactions occurring during regulation of transcription or cellular signaling [36]. Our NMR experiments suggest that TRP120-1TR binds in the micromolar range, much weaker than the low nanomolar dissociation constants (*K*_*d*_) measured for zinc finger transcription factors [37]. Since TRP120 contains multiple TR units, determining whether the additional TR units increase the specificity of DNA targets or DNA binding affinity would require further examination.

Ehrlichial TRPs share several qualities with another group of pathogen effector proteins called TAL effectors, which are secreted by *Xanthomonas* species, and utilize similar transcriptional reprogramming strategies to hijack the host cell. Like the TRPs secreted by *E*. *chaffeensis*, TAL effectors translocate to the host cell nucleus and alter gene regulation to overcome host defenses [11, 38]. The TAL effectors also contain an N-terminal domain, central TR domain, and C-terminal domain. The TRs of ~34 residues contained within TAL effectors are nearly identical, with two varying residues conferring DNA binding specificity for each repeat [39]. Despite the similar domain organization and functional similarities between the TRPs secreted by *E*. *chaffeensis* and the TAL effectors of *Xanthomonas*, significant structural differences exist. Crystal structures of the TR regions from the TAL effectors dHax3 and PthXo1 bound to a DNA target showed that each repeat forms two short α-helices with a flanking loop, and multiple TR units form a right-handed superhelical assembly that wraps around the DNA helix [40, 41]. Notably, in the absence of a DNA target, TAL effectors retain their superhelical assembly [40]. This differs significantly from TRP120, which exists as an intrinsically disordered protein until a coupled DNA binding/folding process occurs.

TRP120 is also different from many DNA-binding proteins that contain a basic surface-exposed patch to complement their negatively charged cognate DNA at the site of interaction [42]. Although each TR unit in TRP120 contains 1 Arg and 5 Lys residues, the sequence of TRP120 is highly acidic overall with a predicted pI value of 4.3 for a single TR unit. TRP120-1TR binds DNA at pH 5.5, but not at pH 7.0, suggesting that acidic pH is required for interaction with DNA. The low pH would decrease the net negative charge on the protein, facilitating interaction with negatively charged DNA. Additionally, because the imidazole side chain has a pKa value within this range (~6.5), His residues are particularly sensitive to pH changes around physiological pH. In some DNA-binding proteins, His residues are known to act as a pH-sensitive trigger, thus this may be relevant because TRP120-1TR contains three His residues (Fig 1A). Such changes in the protonation state of critical His residues can enhance DNA binding affinity without a change in the overall structure of the protein or secondary structure composition. For example, forkhead box P2 protein (FOXP2) is a transcription factor in which pH changes (acidification) increased DNA binding affinity without a change in the secondary structure of the protein in the presence or absence of DNA [43]. The DNA-binding activity can also be enhanced by deprotonation of a critical His residue. The transcription factor early growth response protein 1 (EGR1) undergoes an enhancement in its DNA-binding activity by more than an order of magnitude when a conserved His residue is deprotonated as the pH is raised from 5 to 8 [44]. Alternatively, TRP120 may recruit host or bacterial proteins for its transcriptional activator function to stabilize what would otherwise be a repulsive interaction between acidic TRP120 and DNA. Additional studies on the role of TR units in the context of the whole protein and crosstalk between the TR units and between TR units and the N-terminal and C-terminal domains are necessary to better understand the novel role of TRP120 in the pathology of *E*. *chaffeensis* infection.
